# Serum-Creatinine-to-Cystatin C-to-Waist-Circumference Ratios as an Indicator of Severe Airflow Limitation in Older Adults

**DOI:** 10.3390/jcm12227116

**Published:** 2023-11-15

**Authors:** Jinxuan Li, Qi Sun, Hongguang Zhang, Bingjie Li, Chaoyu Zhang, Yixin Zhao, Jianbo Lu, Xu Ma

**Affiliations:** 1 China-Japan Friendship School of Clinical Medicine, Capital Medical University, Beijing 100029, China; lijx@mail.ccmu.edu.cn; 2 National Center for Respiratory Medicine, State Key Laboratory of Respiratory Health and Multimorbidity, National Clinical Research Center for Respiratory Diseases, Institute of Respiratory Medicine, Chinese Academy of Medical Sciences, Department of Pediatrics, China-Japan Friendship Hospital, Beijing 100029, China; cmusunqi@163.com; 3 National Human Genetic Resources Center, National Research Institute for Family Planning, Beijing 100081, China; zhg@e-health.org.cn (H.Z.); bingjieli1996@163.com (B.L.); zhangchaoyu@nrifp.org.cn (C.Z.); zhaoyixin@nrifp.org.cn (Y.Z.); 4 Graduate School of Peking Union Medical College, Chinese Academy of Medical Sciences, Beijing 100730, China

**Keywords:** peak expiratory flow, lung function, severe airflow limitation, abdominal obesity, sarcopenia

## Abstract

Background: This study aimed to investigate the association between the serum-creatinine-to-cystatin C-to-waist-circumference (CCR/WC) ratio with lung function and severe airflow limitation (SAL). Methods: The data were derived from the China Health and Retirement Longitudinal Study. Peak expiratory flow (PEF) was used as a measure of lung function parameter. Logistic and linear regression were utilized separately to evaluate the relationship between the CCR/WC ratio with PEF and SAL in baseline. Restricted cubic spline was used to explore potential non-linear associations between the CCR/WC ratio and SAL. Cox proportional-hazards models were used to assess the association between CCR/WC quartiles and the risk of new-onset SAL. Results: A total of 6105 participants were included. This study revealed a positive association between the CCR/WC ratio and lung function (PEF: β [partial coefficient]: 25.95, 95%CI: 12.72 to 39.18, *p* < 0.001; PEF/PEF _prediction_: β = 0.08, 95%CI: 0.05 to 0.12, *p* < 0.001) and an inverse association relationship with SAL (OR [odds ratio]: 0.64, 95% confidence interval [CI]: 0.47 to 0.85, *p* = 0.003). Subgroup analysis showed a significant association between the CCR/WC ratio and SAL in males (OR: 0.58, 95% CI: 0.37 to 0.90, *p* = 0.017) but not in females (*p* = 0.059). Cox regression analysis revealed a decreased risk of SAL in the quartiles (Q2–4) compared to the first quartile of the CCR/WC ratio (hazard ratios [HRs]: 0.49 to 0.73, all *p* < 0.05). Conclusions: This study highlights a positive association between the CCR/WC ratio and lung function, with a potential protective effect against SAL.

## 1. Introduction

The rapid development of socio-economic conditions has led to a further deepening of population aging in the China [1]. The significant growth in the elderly population will considerably increase the burden of diseases and healthcare demands on society [2]. Among these concerns, respiratory health in the elderly has attracted increasing attention. Lung function gradually declines with age, affecting the incidence and mortality rates of various respiratory diseases [3]. Peak expiratory flow (PEF) is an accessible index of lung function and has been reported as one of the most important predictors of adverse health outcomes in older adults [4,5]. Thus, investigating the decline in lung function and the occurrence of respiratory limitations in the elderly holds crucial public health significance.

Some research evidence suggests that the decline in lung function may be associated with sarcopenia [6]. Sarcopenia is a common age-related condition characterized by gradual loss of skeletal muscle mass and muscle function, which can significantly lead to a range of health-related adverse outcomes [7]. Similarly, the decline in respiratory muscle strength and function that occurs with aging has been referred to as “respiratory muscle atrophy” [8]. Previous studies have found an association between sarcopenia in patients with chronic lung diseases and poor lung function, which negatively impacts clinical prognosis [9]. Furthermore, longitudinal changes in skeletal muscle mass have been significantly correlated with declines in lung function in otherwise healthy individuals [10]. These studies suggest a certain association between the decline in lung function and muscle function.

The creatinine-to-cystatin C ratio (CCR), known as a biomarker of muscle mass, serves as an alternative indicator of sarcopenia [11]. Creatinine is a product of muscle metabolism, primarily influenced by skeletal muscle [12]. Cystatin C, on the other hand, is a small protein produced by all nucleated cells in the body [13]. CCR is not only a valuable indicator of declines in skeletal muscle mass and strength but also of respiratory system diseases. The creatinine-to-cystatin C ratio is significantly associated with forced vital capacity and forced expiratory volume in one-second values [14]. It is also correlated with acute exacerbations in chronic obstructive pulmonary disease [15]. Furthermore, CCR has demonstrated predictive capability for adverse health outcomes in older adults, including cardiovascular events [16], hospitalizations [17], and even mortality [18]. Recent studies have demonstrated that central obesity and lifestyle have a significant effect on lung function [19,20]. As a result, when evaluating the risk of lung function, relying solely on estimating the CCR is inadequate, and the influence of obesity should be carefully considered. Waist circumference, which serves as an indicator of obesity, proves to be more suitable for assessing obesity in Asian populations compared to body mass index (BMI) [21]. Given the consideration of the CCR metric to assess participants’ muscle mass and WC as an indicator of obesity, in this study, we employ the CCR/WC ratio to provide a comprehensive evaluation of both muscle mass and obesity among the participants.

Prior investigations have reported an association between the CCR/WC ratio and the development of diabetes [22]. However, there is currently a lack of studies examining the correlation between the CCR/WC ratio and lung function in the elderly population. Therefore, the primary objective of this study was to investigate the relationship between the CCR/WC ratio and respiratory impairment in elderly Chinese residents, utilizing a cross-sectional and longitudinal study design.

## 2. Materials and Methods

### 2.1. Study Population

**Figure 1:**
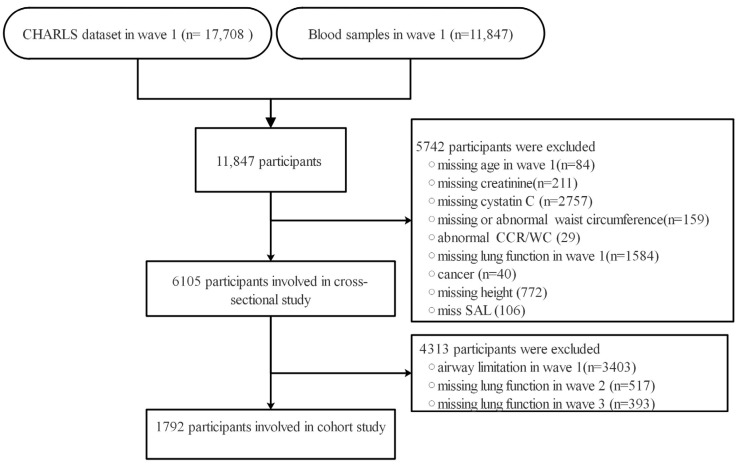
The workflow of study. SAL: severe airflow limitation; CHARLS: China Health and Retirement Longitudinal Study; CCR/WC: serum-creatinine-to-cystatin C-to-waist-circumference ratio.

The data utilized in this study were derived from the China Health and Retirement Longitudinal Study (CHARLS), a comprehensive longitudinal research project. The CHARLS encompassed 10,257 households and involved 17,708 middle-aged and elderly participants from 450 villages or urban communities across 150 counties or districts in 28 provinces, and detailed information of the sampling design has been previously published [23]. The baseline survey was conducted in 2011–2012, and 17,708 participants were enrolled for follow-up assessments at two-year intervals. From 2011 to 2018, the participants underwent 4 waves of follow-up through face-to-face individual interviews, each occurring every two years. As lung function assessments were conducted in 3 waves (in 2011, 2013, and 2015), we utilized the data from these 3 waves of the CHARLS: Wave 1 (2011), Wave 2 (2013), and Wave 3 (2015). This study’s workflow is presented in Figure 1.

This study consists of two parts, with the first part adopting a cross-sectional design to explore the relationship between the CCR/WC ratio and lung function. Participants were excluded based on the following criteria: (1) missing age in wave 1 (*n* = 84), (2) missing creatinine (*n* = 211), (3) missing cystatin C (*n* = 2757), (4) missing or abnormal waist circumference (*n* = 159), (5) abnormal CCR/WC (*n* = 29), (6) missing lung function data in wave 1 (*n* = 1584), (7) history of cancer (*n* = 40), (8) missing height (*n* = 772), or (9) missing severe airflow limitation (SAL) data (*n* = 106). Ultimately, a total of 6105 participants were eligible for inclusion in the cross-sectional analysis. The comparison of baseline information between excluded and included participants is presented in Supplementary Table S1. The second part of the study adopted a cohort study design with the objective of investigating the relationship between the CCR/WC ratio and the risk of new-onset SAL. Individuals with airflow limitation at baseline and participants with missing lung function data in waves 2 and 3 were excluded. A total of 1792 participants were included in the cohort study.

The research design of the CHARLS was approved by the Ethics Review Committee at Peking University (IRB 00001052-11014). Informed consent was obtained from all participants before the interviews commenced.

### 2.2. Exposure Measurement

Blood samples were collected after an overnight fasting period. The measurement of creatinine was performed using the rate-blanked and compensated Jaffe creatinine method, while the measurement of cystatin C was conducted using the particle-enhanced turbidimetric immunoassay. Detailed assay procedures can be found in the CHARLS Blood Test Data User Manual (https://charls.charlsdata.com/index/zh-cn.html, accessed on 20 July 2023). The waist circumference was measured by placing a tape measure horizontally around the participant’s waist at the level of the umbilicus, with the participant standing still, breathing calmly, and holding their breath at the end of expiration. The CCR (creatinine clearance rate) is calculated by dividing the creatinine value (mg/dL) by the cystatin C value (mg/L) and then multiplying the result by 100. The CCR/WC ratio is obtained by dividing the creatinine-to-cystatin C ratio by the waist circumference in centimeters (cm) [22].

### 2.3. Lung Function Assessment

In the CHARLS, lung function was assessed using a peak flow meter. Participants were instructed to stand upright, take a deep breath, and align their mouth with a one-time-use mouthpiece. They were then asked to exhale with maximal force and at the fastest speed possible. Three measurements were recorded, and the highest value among the three breath maneuvers was utilized for data analysis. The predicted peak expiratory flow (PEF _prediction_) was calculated using the following formulas [24]: For males: 75.6 + 20.4 × age—0.41 × age^2^ + 0.002 × age^3^ + 1.19 × height (cm). For females: 282.0 + 1.79 × age—0.046 × age^2^ + 0.68 × height (cm). An airflow limitation was defined as PEF/PEF _prediction_ < 80%, while SAL was defined as PEF/PEF _prediction_ < 60% [25].

### 2.4. Covariates

This study included several covariates: age (years), sex (male/female), residence (urban community/rural village), marital status (married/unmarried), educational level (less than lower secondary/upper secondary and vocational training/tertiary), smoking status (yes/no), drinking (yes/no), BMI (kg/m^2^), plasma glucose, total cholesterol (TC), triglycerides (TG), low-density lipoprotein (LDL-C), high-density lipoprotein (HDL-C), glycated hemoglobin A1c (HbA1c), uric acid (UA), and activities of daily living (ADL). ADL was defined as self-reported difficulties or the need for assistance in at least one event, including dressing (yes/no), bathing (yes/no), eating (yes/no), getting in and out of bed (yes/no), toileting (yes/no), and controlling bowel movements (yes/no). Furthermore, the study assessed the presence of various chronic diseases, including hypertension (yes/no), diabetes (yes/no), lung disease (yes/no), cardiovascular disease (yes/no), stroke (yes/no), kidney disease (yes/no), and asthma (yes/no). Lung disease was defined as the presence of chronic lung conditions, such as chronic bronchitis or emphysema, excluding tumors and cancer. BMI was further classified into 4 categories based on Asian criteria: 1. underweight (BMI < 18.5), 2. normal weight (18.5 ≤ BMI < 23), 3. overweight (23 ≤ BMI < 25), and 4. obesity (BMI ≥ 25) [26].

## 3. Statistical Analysis

Continuous variables with a normal distribution were reported as mean ± standard deviation (SD), whereas categorical variables were presented as counts (percentages). The normality of variables was evaluated using the Kolmogorov–Smirnov test or quantile–quantile plot. Differences in continuous variables between SAL and controls were assessed using either the t-test or Wilcoxon test. Differences in categorical variables between SAL and controls were evaluated using the chi-square test or Fisher’s test.

In the cross-sectional analysis at baseline, logistic regression models were employed to investigate the association between the CCR/WC ratio and SAL. Adjustments for confounding factors were made, and odds ratios (ORs) along with their corresponding 95% confidence intervals (CIs) were calculated. Moreover, multivariable linear regression models were employed to investigate the associations between the CCR/WC ratio and PEF, as well as PEF/PEF _prediction_. Partial regression coefficients (β) and their corresponding 95% CIs were used to quantify the strength of the relationships. To identify possible non-linear associations between the CCR/WC ratio and SAL, a restricted cubic spline (RCS) analysis was conducted, with an aim to detect potential inflection points. RCS analysis is a non-linear modeling technique commonly employed to uncover non-linear relationships or threshold effects between variables [27]. This method accomplishes this by fitting a series of piecewise linear regression models that approximate the actual relationship through a combination of piecewise linear functions, thereby identifying potential thresholds or inflection points. Sensitivity analyses were performed by excluding patients with baseline kidney disease, chronic lung diseases, and asthma, to reassess the relationship between the CCR/WC ratio with lung function and SAL.

For the cohort study, only individuals without airflow limitation were included. Participants were categorized into four groups based on their CCR/WC ratio quantiles: Q1 (≤25%), Q2 (25–50%), Q3 (50–75%), and Q4 (>75%). Participants were then followed up, and the occurrence of SAL was recorded during the second and third follow-ups. Survival analysis was conducted using the Kaplan–Meier method, and the “survival” R package (version: 3.4.0) was used for the analysis. Survival results were visually presented using “survminer” R package (version: 0.4.9). Cox proportional-hazards models were employed to analyze the relationship between the CCR/WC ratio and risk of SAL, while controlling for potential confounding factors. The Cox proportional-hazards model was utilized for the analysis of survival data or time to event occurrence. The risks were expressed using hazard ratios (HRs) and their corresponding 95% CIs.

All statistical analyses were conducted using R (version 4.1.0, available from: https://www.r-project.org/, accessed on 20 July 2023). Two-tailed *p* values were used, with a significance level of <0.05 indicating statistical significance.

## 4. Results

### 4.1. Characteristics of Participants

In this study, a total of 6105 participants were included for baseline cross-sectional analysis, and their baseline characteristics were summarized in Table 1. The mean age of the participants was 59.52 ± 9.78 years. Among them, 45.9% were female, and 33.6% were urban residents. At baseline, the PEF in the control group was significantly higher compared to the case group (mean: 342.94 ± SD: 100.52 vs. 161.96 ± 57.59, *p* < 0.001). The mean CCR/WC ratio in the SAL group was lower compared to the control group (mean: 0.92 ± SD: 0.24 vs. 0.94 ± 0.23, *p* = 0.001).

### 4.2. Association between CCR/WC Ratio and Lung Function at Baseline

Table 2 presents the association of the CCR/WC ratio with lung function and SAL at baseline. In all participants, there were significant associations between the CCR/WC ratio and PEF (β: 106.32, 95%CI: 93.30 to 119.35, *p* < 0.001) as well as PEF/PEF _prediction_ (β: 0.05, 95%CI: 0.02 to 0.08, *p* = 0.002) in the crude model. Additionally, the crude model showed an OR of 0.66 (95% CI: 0.52 to 0.84, *p* = 0.001) for SAL. These associations were consistently observed in Adjusted Model 1 (PEF: β = 24.78, 95%CI: 11.96 to 37.59, *p* < 0.001; PEF/PEF _prediction_: β = 0.08, 95%CI: 0.04 to 0.11, *p* < 0.001; SAL: OR = 0.69, 95% CI: 0.52 to 0.92, *p* = 0.011) and Adjusted Model 2 (PEF: β = 25.95, 95%CI: 12.72 to 39.18, *p* < 0.001; PEF/PEF _prediction_: β = 0.08, 95%CI: 0.05 to 0.12, *p* < 0.001; SAL: OR = 0.64, 95% CI: 0.47 to 0.85, *p* = 0.003). The associations remained consistent when analyzing the data separately for males and females, except for the female population, where there was no significant association between the CCR/WC ratio and SAL. Sensitivity analysis yielded similar results when excluding participants with baseline kidney disease, chronic lung disease, or asthma (Supplementary Table S2).

**Figure 2:**
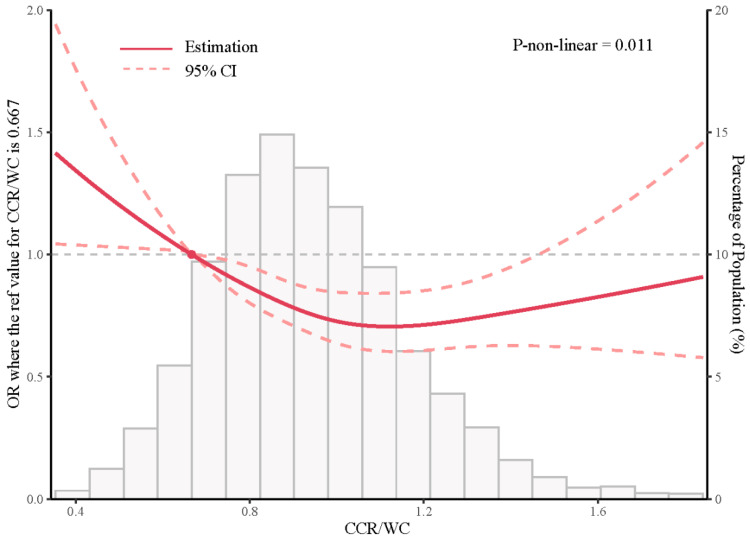
Restricted cubic spline analysis of CCR/WC and SAL. CCR/WC: serum-creatinine-to-cystatin C-to-waist-circumference ratio.

To explore the potential non-linear relationship and the threshold point between the CCR/WC ratio and SAL, restricted cubic spline analysis was applied. The results revealed an L-shaped curve (*p*-non-linear = 0.011) between the CCR/WC ratio and SAL. As the CCR/WC ratio increased, the OR decreased, indicating a protective effect of the CCR/WC ratio for SAL when the CCR/WC ratio was above 0.667 (Figure 2).

### 4.3. Risk of New-Onset SAL of CCR/WC Quartiles

**Figure 3:**
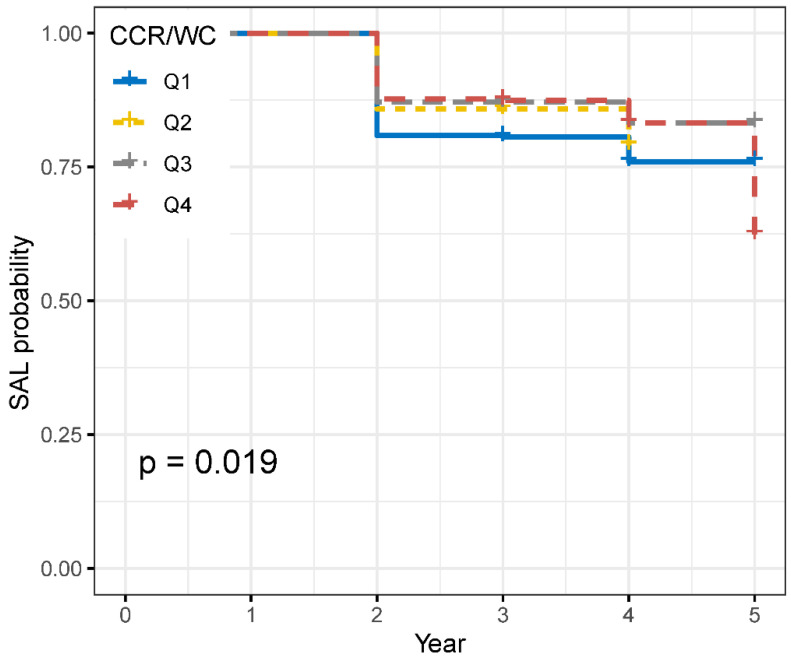
Survival curves of CCR/WC grouped by quartiles. SAL: severe airflow limitation; CCR/WC: serum-creatinine-to-cystatin C-to-waist-circumference ratio. The *p* value was calculated by the log-rank test.

A total of 1792 participants without airflow limitation at baseline were included in this longitudinal analysis to investigate the association between the CCR/WC ratio and the development of new SAL. The CCR/WC ratio was categorized into four groups based on quartiles. Among the 1792 participants, follow-up revealed that 350 individuals experienced SAL events. The survival analysis revealed significant differences in survival rates among the different CCR/WC groups (Figure 3, *p* = 0.019).

Subsequently, Cox proportional-hazards models were employed while controlling for confounding factors to assess the relationship between CCR/WC quartiles and the risk of SAL. Compared to the first quartile, the second quartile showed an HR of 0.73 (95% CI: 0.54 to 0.99, *p* = 0.043), the third quartile had an HR of 0.57 (95% CI: 0.41 to 0.79, *p* = 0.001), and the fourth quartile had an HR of 0.49 (95% CI: 0.34 to 0.70, *p* < 0.001) for the risk of SAL. Similar results were observed in the male group; however, in the female group, after adjusting for confounding factors, this association became non-significant (Table 3).

## 5. Discussion

A population-based study involving elderly individuals in China was conducted, comprising both cross-sectional and longitudinal cohort designs, aiming to investigate the association between the CCR/WC ratio and lung function, as well as SAL. The study findings revealed a positive correlation between the CCR/WC ratio and lung function parameters, including PEF and PEF/PEF _prediction_. Furthermore, logistic regression analysis indicated that the CCR/WC ratio was a protective factor for SAL, and these results were further validated through longitudinal analysis. However, in females, subgroup analysis yielded non-significant results. Additionally, restricted cubic spline analysis demonstrated a non-linear relationship between the CCR/WC ratio and SAL.

To the best of our knowledge, this is the first study to investigate the relationship between the CCR/WC ratio and the risk of SAL. CCR is considered an index of sarcopenia and serves as a surrogate measure of muscle mass. Prior research has indicated that higher standardized CCR is associated with a reduced risk of severe respiratory impairment [28]. However, these studies did not account for the independent effects of muscle mass and fat mass measurements when assessing the risk of airflow limitation [29]. Earlier investigations have also demonstrated that abdominal obesity is linked to an elevated risk of airflow obstruction [30]. Moreover, obesity has been found to lead to impaired respiratory muscle strength and compromised lung function, potentially increasing the risk of respiratory-related chronic diseases [31]. Therefore, in this study, we utilized the CCR/WC ratio to distinguish between muscle mass and abdominal obesity. Our findings revealed that individuals with higher CCR/WC ratios had a lower risk of developing SAL.

To address potential reverse causation in cross-sectional studies, we conducted a follow-up study involving individuals without existing airflow limitation. The results of this longitudinal analysis confirmed the causal relationship between the CCR/WC ratio and SAL. As a novel integrated index of abdominal obesity and muscle mass, this metric has previously been validated as a predictor of type 2 diabetes mellitus incidence [22]. CCR/WC ratios show promise as potential predictive markers for diseases influenced by the combined effects of muscle mass and obesity and could potentially be validated in various other conditions in the future.

The results of subgroup analysis, comprising both cross-sectional and cohort studies, demonstrated that in the male group, the CCR/WC ratio acted as a protective factor for SAL. However, the absence of this association in the female group may be attributed to differences in body composition [32]. Men and women typically exhibit distinct body compositions, leading to variations in fat distribution and muscle mass [33]. These differences may potentially impact the relationship between the CCR/WC ratio and respiratory function in each sex. The disparities in body composition between males and females may be influenced by hormones, lifestyle, and menopause in females [34,35]. Therefore, when using CCR/WC as a biomarker, it is important to consider sex differences. Nevertheless, further research is warranted to investigate whether the relationship between different body compositions and respiratory function is influenced by sex.

The study findings revealed a non-linear relationship between the CCR/WC ratio and the risk of SAL, as evidenced by the restricted cubic spline analysis. This intriguing observation suggests the existence of a critical point where the effect of the CCR/WC ratio on respiratory impairment undergoes a change. One plausible explanation for this threshold effect is that the CCR/WC ratio may act as an indicator of the balance between skeletal muscle mass and abdominal obesity [22]. When the CCR/WC ratio exceeds the threshold value of 0.667, individuals with a higher CCR/WC ratio may possess a relatively higher proportion of muscle mass compared to abdominal fat, which could be associated with better respiratory function. Additionally, the threshold effect might be related to metabolic changes associated with the transition from a healthy to an unhealthy body composition profile. Above the threshold, individuals may exhibit a more favorable body composition with a healthier distribution of muscle and fat, potentially leading to better respiratory outcomes. On the other hand, below the threshold, metabolic alterations associated with obesity-related complications, such as diabetes [22] and cardiovascular disease [36], may be more prominent, potentially exerting a negative impact on respiratory function. Further research is warranted to elucidate the underlying mechanisms and clinical implications of this non-linear relationship between the CCR/WC ratio and respiratory impairment.

The potential mechanisms underlying the association between the CCR/WC ratio and incident airflow limitation are multifaceted. Firstly, the CCR/WC ratio may serve as a representative of overall muscle function [37]. Higher CCR/WC values may indicate better muscle quality and strength, which can positively influence respiratory muscle function and contribute to improved lung mechanics and airflow [38]. Secondly, obesity and changes in body composition can trigger chronic low-grade inflammation [39], which is associated with respiratory diseases. The CCR/WC ratio may reflect the systemic inflammation levels, and chronic inflammation can promote the development of airflow limitation. Thirdly, abdominal obesity, as indicated by the CCR/WC ratio, is often associated with insulin resistance [40]. Insulin resistance is a risk factor for several chronic diseases, including respiratory disorders, and may lead to impaired lung function [41]. Lastly, adipose tissue, particularly visceral fat, secretes various bioactive molecules called adipokines [42]. Dysregulation of adipokines due to obesity could influence lung function and contribute to the development of airflow limitation. These potential mechanisms shed light on the complex interplay between body composition, inflammation, and metabolic factors in the context of respiratory impairment and highlight the importance of considering the CCR/WC ratio as a valuable metric in understanding respiratory health. However, further research is required to fully elucidate the specific pathways and causality of these associations.

Our study has several limitations that need to be acknowledged. Firstly, despite controlling for numerous confounding factors in the analysis, there may still exist some unconsidered confounders that could potentially influence the association between the CCR/WC ratio and respiratory function, such as dietary habits and physical activity frequency. Secondly, our study initially established the association between the CCR/WC ratio and respiratory function using a cross-sectional study design and subsequently validated this association using a cohort design. However, the study design lacks sufficient causal inference capability, and future research should consider employing randomized controlled trials, biological mechanism studies, and intervention trials to establish causal relationships. Thirdly, due to the CHARLS cohort’s limited data collection, only the lung function parameter PEF was available, while other lung function indicators such as forced vital capacity and forced expiratory volume in one second were not collected. Therefore, future research should explore the association between the CCR/WC ratio and other respiratory function indicators to gain a more comprehensive understanding. Fourthly, our study involves interval censoring, meaning that the exact timing of SAL occurrence cannot be precisely determined. The nature of this interval censoring may have an impact on the interpretation of certain data analysis methods and results. Lastly, we utilized 4-year follow-up data from the CHARLS cohort. Future studies should consider larger sample sizes, diverse ethnic populations, and longer-duration multi-center cohort studies to validate and replicate the results of this study.

Despite these limitations, our study provides valuable insights into the relationship between the CCR/WC ratio and respiratory health in the elderly population. For clinical practice, we recommend that healthcare providers should monitor the CCR/WC ratio to prevent declines in lung function among older adults. Additionally, we suggest that policymakers should formulate relevant public health policies aimed at enhancing lung function health and preventing respiratory diseases.

## 6. Conclusions

Our study yields valuable insights into the relationship between the CCR/WC ratio and lung function in Chinese adults. We observed a positive correlation between the CCR/WC ratio and lung function, indicating that individuals with higher CCR/WC ratios may exhibit better respiratory outcomes. Additionally, our findings suggest a potential protective effect of the CCR/WC ratio against SAL, suggesting that monitoring and improving the CCR/WC ratio could be a relevant approach in the prevention of respiratory impairment. These results underscore the importance of considering body composition factors, such as the CCR/WC ratio, in respiratory health assessments and interventions, potentially offering new avenues for improving respiratory outcomes in the adult population.

## Figures and Tables

**Table 1:** Characteristics of the participants at baseline.

Variables		Overall	Controls (*n* = 4259)	SAL (*n* = 1846)	*p*
Age (years)		59.52 ± 9.78	58.90 ± 9.68	60.95 ± 9.85	<0.001
Sex	Male	2804 (45.9)	1965 (46.1)	839 (45.4)	0.640
	Female	3301 (54.1)	2294 (53.9)	1007 (54.6)	
Residence	Urban Community	2049 (33.6)	1463 (34.4)	586 (31.7)	0.051
	Rural Village	4056 (66.4)	2796 (65.6)	1260 (68.3)	
Married	Unmarried	1026 (16.8)	692 (16.2)	334 (18.1)	0.083
	Married	5079 (83.2)	3567 (83.8)	1512 (81.9)	
Educational	Less than lower secondary	5563 (91.1)	3831 (90.0)	1732 (93.8)	<0.001
	Upper secondary and vocational training	475 (7.8)	370 (8.7)	105 (5.7)	
	Tertiary	67 (1.1)	58 (1.4)	9 (0.5)	
Smoking	Never smoked	3742 (61.5)	2651 (62.4)	1091 (59.5)	0.027
	Former smoker	517 (8.5)	338 (8.0)	179 (9.8)	
	Current smoker	1825 (30.0)	1260 (29.7)	565 (30.8)	
Drinking	No	3748 (61.4)	2633 (61.8)	1115 (60.4)	0.315
	Yes	2355 (38.6)	1625 (38.2)	730 (39.6)	
BMI (kg/m^2^)	<18.5	427 (7.0)	229 (5.4)	198 (10.8)	<0.001
	18.5 to 23.9	2547 (41.9)	1736 (40.9)	811 (44.2)	
	23 to 24.9	1242 (20.4)	893 (21.0)	349 (19.0)	
	25 to 100	1862 (30.6)	1387 (32.7)	475 (25.9)	
PG (mg/dL)		110.07 ± 35.31	110.20 ± 34.33	109.76 ± 37.48	0.652
TC (mg/dL)		193.33 ± 38.65	194.16 ± 38.33	191.42 ± 39.31	0.011
TG (mg/dL)		131.28 ± 98.55	132.84 ± 99.30	127.71 ± 96.72	0.062
LDL-C (mg/dL)		116.52 ± 35.19	118.05 ± 35.02	113.00 ± 35.35	<0.001
HDL-C (mg/dL)		51.20 ± 15.18	50.69 ± 14.85	52.40 ± 15.85	<0.001
HbA1c (mg/dL)		5.27 ± 0.80	5.25 ± 0.78	5.29 ± 0.83	0.074
UA (mg/dL)		4.44 ± 1.26	4.46 ± 1.26	4.40 ± 1.27	0.092
Creatinine (mg/dL)		0.78 ± 0.24	0.78 ± 0.20	0.77 ± 0.32	0.062
Cystatin C (mg/L)		1.01 ± 0.27	1.01 ± 0.25	1.04 ± 0.32	<0.001
CCR/WC		0.94 ± 0.23	0.94 ± 0.23	0.92 ± 0.24	0.001
ADL	No	5061 (83.7)	3641 (86.3)	1420 (77.9)	<0.001
	Yes	982 (16.3)	578 (13.7)	404 (22.1)	
Hypertension	No	4466 (73.5)	3153 (74.4)	1313 (71.4)	0.016
	Yes	1610 (26.5)	1084 (25.6)	526 (28.6)	
Diabetes	No	5690 (93.8)	3953 (93.5)	1737 (94.4)	0.239
	Yes	378 (6.2)	274 (6.5)	104 (5.6)	
Lung disease	No	5472 (89.9)	3960 (93.2)	1512 (82.1)	<0.001
	Yes	616 (10.1)	287 (6.8)	329 (17.9)	
CVD	No	5320 (87.5)	3754 (88.5)	1566 (85.3)	0.001
	Yes	758 (12.5)	488 (11.5)	270 (14.7)	
Stroke	No	5953 (97.8)	4167 (98.1)	1786 (97.1)	0.019
	Yes	136 (2.2)	82 (1.9)	54 (2.9)	
Kidney	No	5716 (94.2)	4015 (94.8)	1701 (92.7)	0.003
	Yes	355 (5.8)	222 (5.2)	133 (7.3)	
Asthma	No	5794 (95.2)	4133 (97.4)	1661 (90.3)	<0.001
	Yes	290 (4.8)	112 (2.6)	178 (9.7)	
PEF (L/min)		288.22 ± 122.32	342.94 ± 100.52	161.96 ± 57.59	<0.001

The CCR/WC is calculated as (Creatinine/Cystatin C) × 100/waist circumference. PG: plasma glucose; TC: total cholesterol; TG: triglycerides; LDL-C: low-density lipoprotein cholesterol; HDL-C: high-density lipoprotein cholesterol; HbA1c: glycated hemoglobin A1c; UA: uric acid; SAL: severe airflow limitation; BMI: body mass index; PEF: peak expiratory flow; ADL: activities of daily living; CVD: cardiovascular disease.

**Table 2:** Association of CCR/WC with lung function and SAL.

		PEF		PEF/PEF _prediction_		SAL	
		β (95%CI)	*p*	β (95%CI)	*p*	OR (95%CI)	*p*
All							
	Crude model	106.32 (93.30, 119.35)	<0.001	0.05 (0.02, 0.08)	0.002	0.66 (0.52, 0.84)	0.001
	Adjusted model 1	24.78 (11.96, 37.59)	<0.001	0.08 (0.04, 0.11)	<0.001	0.69 (0.52, 0.92)	0.011
	Adjusted model 2	25.95 (12.72, 39.18)	<0.001	0.08 (0.05, 0.12)	<0.001	0.64 (0.47, 0.85)	0.003
Male							
	Crude model	73.82 (51.50, 96.14)	<0.001	0.05 (0.00, 0.10)	0.033	0.53 (0.36, 0.77)	0.001
	Adjusted model 1	26.81 (5.50, 48.12)	0.014	0.07 (0.03, 0.12)	0.003	0.60 (0.39, 0.92)	0.019
	Adjusted model 2	27.86 (5.94, 49.78)	0.013	0.08 (0.03, 0.13)	0.003	0.58 (0.37, 0.90)	0.017
Female							
	Crude model	46.13 (31.55, 60.71)	<0.001	0.05 (0.01, 0.10)	0.013	0.76 (0.54, 1.06)	0.112
	Adjusted model 1	21.50 (6.41, 36.58)	0.005	0.08 (0.03, 0.12)	0.001	0.78 (0.53, 1.15)	0.209
	Adjusted model 2	24.78 (9.10, 40.46)	0.002	0.09 (0.04, 0.14)	<0.001	0.67 (0.45, 1.00)	0.059

PEF: peak expiratory flow; PEF/PEF _prediction_: ratio of peak expiratory flow to predicted peak expiratory flow; SAL: Severe airflow limitation; β: coefficient; OR: odds ratio; CI: confidence interval. Adjusted Model 1: model adjusted for age at baseline, sex, rural or urban residence status, marital status, educational level, smoking status, drinking, body mass index category, activities of daily living, hypertension, diabetes, lung disease, heart disease, stroke, kidney disease, and asthma. Adjusted Model 2: model further adjusted for fasting plasma glucose, total cholesterol, triglycerides, low-density lipoprotein cholesterol, high-density lipoprotein cholesterol, glycosylated hemoglobin, and uric acid.

**Table 3:** Association of CCR/WC with new-onset SAL.

		All		Male		Female	
		HR (95%CI)	*p*	HR (95%CI)	*p*	HR (95%CI)	*p*
Crude model							
	Q1	Ref		Ref		Ref	
	Q2	0.85 (0.64, 1.12)	0.253	0.69 (0.42, 1.14)	0.149	0.93 (0.66, 1.31)	0.663
	Q3	0.67 (0.50, 0.90)	0.008	0.47 (0.28, 0.78)	0.004	0.86 (0.59, 1.26)	0.442
	Q4	0.67 (0.50, 0.91)	0.010	0.61 (0.38, 0.97)	0.037	0.65 (0.41, 1.03)	0.069
Adjusted model 1							
	Q1	Ref		Ref		Ref	
	Q2	0.75 (0.55, 1.01)	0.055	0.56 (0.33, 0.94)	0.029	0.74 (0.51, 1.08)	0.117
	Q3	0.57 (0.41, 0.79)	0.001	0.36 (0.21, 0.62)	0.000	0.66 (0.44, 1.00)	0.049
	Q4	0.49 (0.35, 0.70)	<0.001	0.46 (0.27, 0.77)	0.003	0.41 (0.24, 0.70)	0.001
Adjusted model 2							
	Q1	Ref		Ref		Ref	
	Q2	0.73 (0.54, 0.99)	0.043	0.58 (0.35, 0.99)	0.044	0.73 (0.50, 1.07)	0.104
	Q3	0.57 (0.41, 0.79)	0.001	0.40 (0.23, 0.68)	0.001	0.65 (0.43, 1.00)	0.048
	Q4	0.49 (0.34, 0.70)	<0.001	0.53 (0.31, 0.90)	0.018	0.40 (0.23, 0.69)	0.001

HR: hazard ratio; CI: confidence interval. The CCR/WC ratio was categorized into four quartiles, namely Q1 to Q4. Adjusted Model 1: model adjusted for age at baseline, sex, rural or urban residence status, marital status, educational level, smoking status, drinking, body mass index category, activities of daily living, hypertension, diabetes, lung disease, heart disease, stroke, kidney disease, and asthma. Adjusted Model 2: model further adjusted for fasting plasma glucose, total cholesterol, triglycerides, low-density lipoprotein cholesterol, high-density lipoprotein cholesterol, glycosylated hemoglobin, and uric acid.

## Data Availability

The data that support the findings of this study are available from the corresponding author upon reasonable request.

## Supplementary Materials

The following supporting information can be downloaded at: https://www.mdpi.com/article/10.3390/jcm12227116/s1, Table S1. The comparison of baseline information between excluded and included participants. Table S2. Association of CCR/WC with Pulmonary Function in Populations without Renal Disease, Chronic Pulmonary Disorders, and Asthma.
