# Supplementary material for: Serum-Creatinine-to-Cystatin C-to-Waist-Circumference Ratios as an Indicator of Severe Airflow Limitation in Older Adults
Source: J Clin Med. 2023 Nov 15;12(22):7116. doi: 10.3390/jcm12227116 (PMC10672224; doi:10.3390/jcm12227116)
Supplement: Supplementary file 1 [file jcm-12-07116-s001.zip › jcm-2603333-supplementary.pdf]

Supplemental Table S1. The comparison of baseline information between excluded and included participants.

	level	Overall (n = 11847)	Excluded (n = 5742)	Included (n = 6105)	*P*
Age (years)		58.60 ± 9.81	57.60 ± 9.74	59.52 ± 9.78	< 0.001
Sex	Male	5503 (46.5)	2699 (47.0)	2804 (45.9)	0.248
	Female	6344 (53.5)	3043 (53.0)	3301 (54.1)	
Residence	Urban Community	4317 (36.4)	2268 (39.5)	2049 (33.6)	< 0.001
	Rural Village	7530 (63.6)	3474 (60.5)	4056 (66.4)	
Married	No	2057 (17.4)	1031 (18.0)	1026 (16.8)	0.102
	Yes	9788 (82.6)	4709 (82.0)	5079 (83.2)	
Educational	Less than lower secondary	10640 (89.8)	5077 (88.4)	5563 (91.1)	< 0.001
	Upper secondary & vocational training	1041 (8.8)	566 (9.9)	475 (7.8)	
	Tertiary	164 (1.4)	97 (1.7)	67 (1.1)	
Smoking	Never smoking	7185 (62.1)	3443 (62.8)	3742 (61.5)	0.154
	Former smoking	1000 (8.6)	483 (8.8)	517 (8.5)	
	Current smoking	3380 (29.2)	1555 (28.4)	1825 (30.0)	
Drinking	No	7237 (61.4)	3489 (61.4)	3748 (61.4)	0.979
	Yes	4551 (38.6)	2196 (38.6)	2355 (38.6)	
BMI (kg/m2)	< 18.5	680 (6.8)	253 (6.5)	427 (7.0)	0.136
	18.5 to 23.9	4123 (41.4)	1576 (40.7)	2547 (41.9)	
	23 to 24.9	2014 (20.2)	772 (19.9)	1242 (20.4)	
	25 to 100	3133 (31.5)	1271 (32.8)	1862 (30.6)	
WC (cm)		84.34 ± 12.60	82.99 ± 15.53	85.21 ± 10.19	< 0.001
SBP		129.58 ± 21.58	129.44 ± 21.39	129.67 ± 21.70	0.596
DBP		75.35 ± 12.24	75.85 ± 12.31	75.03 ± 12.19	0.001
PG (mg/dL)		110.30 ± 37.33	110.56 ± 39.45	110.07 ± 35.31	0.480
TC (mg/dL)		192.97 ± 38.89	192.56 ± 39.16	193.33 ± 38.65	0.288
TG (mg/dL)		134.91 ± 110.26	138.89 ± 121.73	131.28 ± 98.55	< 0.001
LDL-C (mg/dL)		115.99 ± 34.91	115.41 ± 34.59	116.52 ± 35.19	0.087
HDL-C (mg/dL)		50.84 ± 15.33	50.45 ± 15.48	51.20 ± 15.18	0.008
HbA1c (mg/dL)		5.26 ± 0.82	5.26 ± 0.85	5.27 ± 0.80	0.694
UA (mg/dL)		4.46 ± 1.27	4.49 ± 1.27	4.44 ± 1.26	0.061
Creatinine (mg/dL)		0.78 ± 0.24	0.79 ± 0.24	0.78 ± 0.24	0.292
Cystatin C (mg/L)		1.02 ± 0.29	1.03 ± 0.32	1.01 ± 0.27	0.004
CCR/WC		0.98 ± 0.41	1.16 ± 0.81	0.94 ± 0.23	< 0.001
ADL	No	9685 (83.0)	4624 (82.2)	5061 (83.7)	0.025
	Yes	1985 (17.0)	1003 (17.8)	982 (16.3)	
Hypertension	No	8548 (72.9)	4082 (72.2)	4466 (73.5)	0.128

	Yes	3179 (27.1)	1569 (27.8)	1610 (26.5)	
Diabetes	No	10953 (93.8)	5263 (93.7)	5690 (93.8)	0.960
	Yes	730 (6.2)	352 (6.3)	378 (6.2)	
Lung disease	No	10598 (90.2)	5126 (90.5)	5472 (89.9)	0.236
	Yes	1151 (9.8)	535 (9.5)	616 (10.1)	
CVD	No	10269 (87.5)	4949 (87.5)	5320 (87.5)	0.999
	Yes	1462 (12.5)	704 (12.5)	758 (12.5)	
Stroke	No	11437 (97.2)	5484 (96.6)	5953 (97.8)	< 0.001
	Yes	329 (2.8)	193 (3.4)	136 (2.2)	
Kidney	No	11030 (94.1)	5314 (94.1)	5716 (94.2)	1.000
	Yes	686 (5.9)	331 (5.9)	355 (5.8)	
Asthma	No	11223 (95.5)	5429 (95.8)	5794 (95.2)	0.178
	Yes	530 (4.5)	240 (4.2)	290 (4.8)	
PEF (L/min)		291.83 (124.25)	297.94 (127.24)	288.22 (122.32)	< 0.001

PG: Plasma glucose; TC: Total cholesterol; TG: Triglycerides; LDL-C: Low-density lipoprotein cholesterol; HDL-C: High-density lipoprotein cholesterol; HbA1c: Glycated hemoglobin A1c; UA: Uric acid; SAL: Severe airflow limitation; WC: Waist circumference; The CCR/WC is calculated as (Creatinine/Cystatin C) * 100/waist circumference; BMI: Body Mass Index; PEF: peak expiratory flow; ADL: activities of daily living; CVD: Cardiovascular disease.

Supplemental Table S2. Association of CCR/WC with Pulmonary Function in Populations without Renal Disease, Chronic Pulmonary Disorders, and Asthma

	β/OR (95%CI)	*P*
SAL	0.63 (0.45, 0.87)	0.005
PEF	25.23 (10.77, 39.69)	0.001
PEF/ $PEF_{prediction}$	0.08 (0.05, 0.12)	< 0.001

SAL: Specific Resistance of the Airway; PEF: Peak Expiratory Flow; $PEF_{prediction}$: Predicted Peak Expiratory Flow; β/OR (95%CI): coefficient /Odds Ratio with 95% Confidence Interval.
